# Hesperidin protects rats’ liver and kidney from oxidative damage and physiological disruption induced by nickel oxide nanoparticles

**DOI:** 10.3389/fphys.2022.912625

**Published:** 2022-10-19

**Authors:** Ahmed Abd-Eltawab Tammam, Abdel Azeim A. Khalaf, Amr R. Zaki, Mohamed Mansour Khalifa, Marwa A. Ibrahim, Aya M. Mekkawy, Rehab E. Abdelrahman, Ahmed Farghali, Peter A. Noshy

**Affiliations:** ^1^ Medical Physiology Department, College of Medicine, Jouf University, Sakaka, Saudi Arabia; ^2^ Medical Physiology Department, Faculty of Medicine, Beni-Suef University, Beni-Suef, Egypt; ^3^ Department of Toxicology and Forensic Medicine, Faculty of Veterinary Medicine, Cairo University, Giza, Egypt; ^4^ Department of Forensic Medicine and Clinical Toxicology, Faculty of Medicine, Beni-Suef University, Beni-Suef, Egypt; ^5^ Department of Human Physiology, Faculty of Medicine, Cairo University, Cairo, Egypt; ^6^ Department of Human Physiology, College of Medicine, King Saud University, Riyadh, Saudi Arabia; ^7^ Department of Biochemistry and Molecular Biology, Faculty of Veterinary Medicine, Cairo University, Giza, Egypt; ^8^ Department of Cytology and Histology, Faculty of Veterinary Medicine, Cairo University, Giza, Egypt; ^9^ Materials Science and Nanotechnology Department, Faculty of Postgraduate Studies for Advanced Sciences, Beni-Suef University, Beni-Suef, Egypt

**Keywords:** flavanone glycoside, hesperidin, kidney, liver, nickel oxide nanoparticles, oxidative stress, toxicity

## Abstract

**Background:** Nickel oxide nanoparticles (NiO-NPs) have recently been utilized in various advanced industrial fields like lithium-ion micro batteries, nanofibers, electrochromic devices, and several biomedical applications. NiO-NPs are classified as extremely toxic substances as they can cause long-term harm to the environment and aquatic life. Moreover, frequent and prolonged exposure can affect human and animal health, causing skin allergies and major toxic consequences, such as hepatorenal toxicity. Hesperidin (HSP) has been proven to possess anti-inflammatory, antioxidant, and free radical scavenging activities.

**Objective:** This study aimed to investigate the underlying protective mechanisms and effects of HSP against NiO-NPs-induced hepatorenal toxicities in rats.

**Materials and Methods:** Forty male Wistar rats were randomly divided into four groups (n = 10 in each). The first group served as a Control group. For 8 weeks, the second group was administered NiO-NPs (100 mg/kg/day), and the third group was given HSP (100 mg/kg/day) *via* oral gavage for both groups. The fourth group received NiO-NPs and HSP concurrently in the same oral daily doses and duration as the second and third groups.

**Results:** NiO-NPs administration revealed a significant increase in plasma biomarkers of nephrotoxicity (urea, creatinine) and hepatotoxicity (ALT, AST) in NiO-NPs group compared to Control group (*p* < 0.05). In addition, NiO-NPs administration resulted in a substantial increase in malondialdehyde levels with a significant drop in catalase activity and GSH content in Group II. Also, a significant decreased expression of Nrf-2 and Bcl-2 mRNA levels and upregulation of TNF-α, NF-kβ and BAX in the liver and kidney of NiO-NPs group were also detected. Histologically, the liver and kidney of rats of NiO-NPs group showed significant histopathological disturbances, with a substantial increase in the proliferating cell nuclear antigen (PCNA) positive hepatocytes and renal tubular cells in the NiO-NPs group compared to Control and HSP groups (*p* < 0.05). In contrast, concomitant administration of HSP with NiO-NPs in group IV showed a significant biochemical, histopathological, and immunohistochemical improvement compared to NiO-NPs group.

**Conclusion:** Co-administration of HSP with NiO-NPs significantly ameliorated most of the NiO-NPs-induced hepatorenal toxicities in male rats.

## Introduction

Nanotechnology is one of the most developing fields of scientific research. Accordingly, recent progressions in biomedicine, electronics, energy and cosmetics are concurrent with the development of nanotechnology (Benavides et al., 2016)**.** Nanoparticles (NPs) are materials with dimensions of less than 100 nm (Morsy et al., 2018). Humans and animals can be exposed to nanomaterials occasionally through oral, inhalation, and dermal routes (Baek et al., 2018)**.** Nanomaterials can be easily absorbed and react with the vital organs (Shaw and Handy, 2011)**.** Nickel oxide NPs (NiO-NPs) are one of the most utilized NPs in a variety of consumer goods such as solid oxide fuel cells, nanowires, catalysts, electrochromic coatings, ceramics, sensors, paints, and storage batteries (Mu et al., 2011), (Siddiqui et al., 2012)**.** The increasing application of NiO-NPs has led to an increase in the environmental load and a dangerous hazard to human and animal health (Cao et al., 2016).

The most exposed organs to Nickel (Ni) are the liver, kidneys, brain, lungs, and testis (Dumala et al., 2018). The main proposed mechanisms of NiO-NPs to induce cytotoxicity are increasing the levels of reactive oxygen species (ROS) (Yu et al., 2018), in addition to stimulating cell degeneration and inflammation (Razavipour et al., 2015). Furthermore, NiO-NPs were found to induce cytogenetic alterations, oxidative stress; and apoptosis (Abudayyak et al., 2017). Magaye et al. (Magaye et al., 2014) reported that nickel nanoparticles cause inflammation in the liver of rats. Moreover, Katsnelson et al. (Katsnelson et al., 2015) reported that nano-NiO administration in rats leads to nickel deposition, increased Kupffer cells, and akaryotic and binucleated hepatocytes in the liver tissues of rats. Nephrotoxicity in response to pollutants can disturb the excretory functions of the kidney and cause alterations in the renal physiology and structure (Zhang and Sun, 2015).

Hesperidin (HSP) is a phytoflavanone glycoside found in citrus fruits like oranges and lemons (Noshy et al., 2022)**.** It possesses many pharmacological effects, including antidiabetic, hepatoprotective, hypolipidemic, anti-inflammatory, anti-carcinogenic; and antioxidant (Choi, 2008)**.** It has been reported that HSP can protect against hepatotoxicity and nephrotoxicity resulting from cisplatin exposure (Omar et al., 2016), (Sahu et al., 2013)**.** The anti-oxidative mechanisms of HSP include enhancing endogenous antioxidant defenses, scavenging free radicals, repressing ROS generating enzymes; and preventing DNA damage (Kumar and Kumar, 2010). Siddiqi et al**.** (Siddiqi et al., 2018) demonstrated the chemo-preventive effect of HSP against nephrotoxicity and renal carcinogenesis. Also, it was shown that HSP could protect many tissues such as the kidney (Sahu et al., 2013), heart (Abdel-Raheem and Abdel-Ghany, 2009) against oxidative damage.

To the best of our knowledge, there is no previous reported study concerning the protective effect of HSP against NiO-NPs-induced liver and kidney toxicities. Therefore, the present study aimed to evaluate the ability of HSP to ameliorate NiO-NPs induced hepatotoxicity and nephrotoxicity in rats by examining various liver and kidney toxicity indicators, including genotoxicity and histopathological and immunohistochemical examinations.

## Materials and methods

### Animals

Forty adult male Wistar rats of the same age (10 weeks) and weighing 200–220 g were obtained from the Research Institute of Ophthalmology, Giza, Egypt. Rats were examined for health status and adapted to the laboratory environment (normal daylight, temperature: 22–28°C) for 10 days before the beginning of the experiment. Rats were reared in plastic cages, fed on pelleted feed, and supplied with water *ad libitum*. The sample size was determined according to statistical hypothesis testing. The experimental design was approved by the Institutional Animal Care and Use Committee at the faculty of veterinary medicine, Cairo University (Vet. CU. IACUC; approval No. 2009-2022512). Rats received humane care in compliance with the National Institutes of Health (NIH) guidelines.

### Preparation of nickel oxide nanoparticles

Nickel oxide nanoparticles (NiO-NPs) were prepared, in the Center of Excellence of Nanotechnology at the Faculty of Postgraduate Study for Advanced Sciences, Beni-Suef University, by precipitating nickel hydroxide from the reaction of sodium hydroxide with nickel acetate. The precipitate was then filtered immediately after complete precipitation, washed well with distilled water, ignited at 600°C for 3 h, and finally milled using the ball milling technique for 10 h to ensure the size reduction and homogeneity. The obtained NiO-NPs fine powder was then characterized using X-Ray Diffraction (XRD) Panalytical Empyrean, Netherlands, Scanning Electron Microscopy (SEM) ZEISS, EVO-MA10; and Transmission Electron Microscopy (TEM) HRTEM, JEM2100, JEOL, Japan.

### Chemicals

Hesperidin (HSP) was obtained from Sigma-Aldrich Co. (United States). NiO-NPs and HSP were suspended in 0.5% carboxymethylcellulose (CMC). All the remaining chemicals and diagnostic kits were procured from Bio diagnostic Co., (Giza, Egypt) and Sigma Chemical Company, St. Louis, USA.

### Experimental design

**FIGURE 1:**
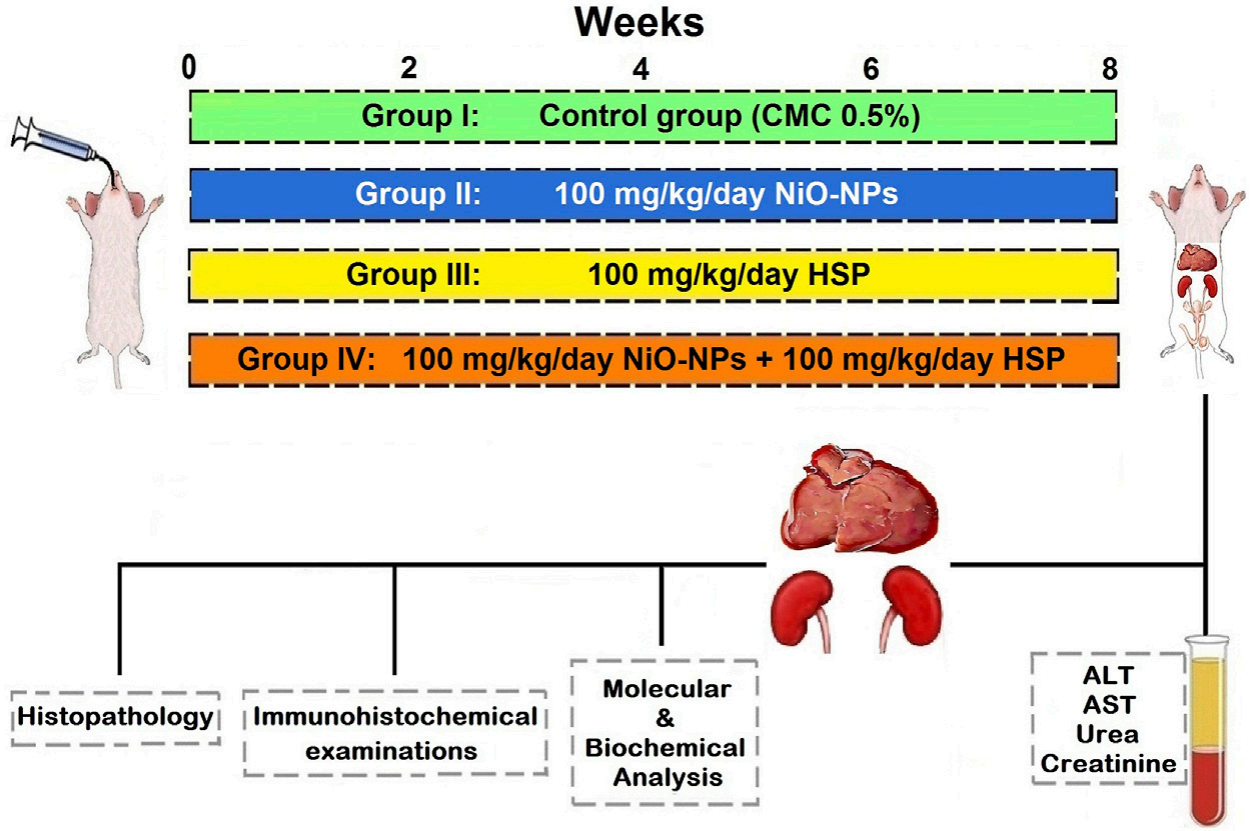
A schematic diagram showing the experimental design.

Rats were randomly divided into four equal groups (10 rats each) which were exposed to daily NiO-NPs and/or HSP doses by oral gavage for 8 weeks as follows (Figure 1):

Group I (Control group): Received CMC 0.5% (the vehicle of NiO-NPs and HSP).

Group II (NiO-NPs group): Received NiO-NPs (100 mg/kg/day) suspended in 0.5% CMC according to previous studies (Dumala et al., 2019), (Ali et al., 2021).

Group III (HSP group): Received HSP (100 mg/kg/day) suspended in 0.5% CMC (Menze et al., 2012), (Noshy and Azouz, 2021).

Group IV (NiO-NPs + HSP group): Received the same aforementioned daily doses of both NiO-NPs and HSP in group II and III respectively with about 6 h interval between NiO-NPs and HSP doses to avoid chelation of NiO-NPs by HSP.

### Collection of samples

After the end of the exposure period, rats were anesthetized using intraperitoneal injection of 90 mg/kg ketamine +10 mg/kg xylazine (in the same syringe). Blood samples were collected from the inner canthus of the eye, followed by centrifugation. The obtained sera were stored at -20°C. Then, rats were euthanized by cervical dislocation to collect tissue samples (liver and kidneys). Hepatic and renal tissue samples were divided into 2 parts; the first part was stored in plastic bags at -20°C for biochemical and molecular investigations, while the other part was fixed in a 10% neutral buffered formalin solution for histopathological and immunohistochemical examinations.

### Liver and kidney function tests

Alanine aminotransferase (ALT), aspartate aminotransferase (AST), urea; and creatinine levels were determined in the serum of rats using kits obtained from Bio-diagnostic Co., Giza, Egypt, following the manufacturer instructions.

### Oxidative stress and lipid peroxidation parameters

Hepatic and renal tissue samples were homogenized in 5 ml cold potassium phosphate buffer (50 mM) per gram tissue using tissue homogenizer followed by centrifugation at 4,000 rpm for 15 min and the supernatant was collected.

Catalase (CAT), reduced glutathione (GSH), and malondialdehyde (MDA) levels were measured in hepatic and renal tissue homogenates using colorimetric kits purchased from Bio-diagnostic Co., Giza, Egypt, following the manufacturer instructions.

### Gene expression analyses

- Total RNA isolation:

Total RNA of different tissues were isolated using RNeasy Mini Kit (Qiagen Cat No./ID: 74104) followed by synthesis of the first-strand cDNA using SuperScript Reverse Transcriptases (Thermo Scientific) according to the manufacturer’s instructions.

- The quantitative real-time PCR:

We have designed our primer sets using primer 3 software and checked them using the NCBI Primer blast tool. All the primers showed efficient amplification for amplicons between 100–300; their melting temperature range between 59–61°C.

The primer sets used to amplify the relative expression levels of the target genes are listed in Table 1. Quantitative PCR was performed using SYBR™ Green PCR Master Mix (Thermo scientific Cat number: 4309155) (Mo et al., 2022)**.** ABI Prism Step One Plus Real-Time PCR System (Applied Biosystems) according to the manufacturer’s instructions. The target mRNA expression was normalized to ACTB (Helmy et al., 2020).

**TABLE 1:** The primer sets used in the study.

	Sense	Antisense	Amplicon	Accession no
*NF-k*β	TTC​AAC​ATG​GCA​GAC​GAC​GA	TGC​TCT​AGT​ATT​TGA​AGG​TAT​GGG	146	NM_001276711.1
*TNF-α*	ACA​CAC​GAG​ACG​CTG​AAG​TA	GGA​ACA​GTC​TGG​GAA​GCT​CT	235	NM_012675.3
*Nrf-2*	TGT​AGA​TGA​CCA​TGA​GTC​GC	TCC​TGC​CAA​ACT​TGC​TCC​AT	159	NM_031789.2
ACTB	CCG​CGA​GTA​CAA​CCT​TCT​TG	CAG​TTG​GTG​ACA​ATG​CCG​TG	297	NM_031144.3
*BAX*	CAC​GTC​TGC​GGG​GAG​TCA​C	TTC​TTG​GTG​GAT​GCG​TCC​TG	248	NM_017059.2
*Bcl-2*	TCG​CGA​CTT​TGC​AGA​GAT​GT	CAA​TCC​TCC​CCC​AGT​TCA​CC	116	NM_016993.2

### Histopathological examination

The fixed samples were dehydrated with a series of alcohol washes, then cleared in xylene, and embedded in paraffin wax. Paraffin sections of 3–4 μm thick were prepared using a rotatory microtome, then tissue sections were deparaffinized and stained with hematoxylin and eosin (H&E) for histopathological studies (Bancroft and Gamble, 2008)**.** Chemicals used in the stain were purchased from Sigma Chemical Company, St. Louis, USA.

### Immuno-histochemical examination

Additional sections were prepared for immunohistochemical analysis. Detection of PCNA in the liver and kidney tissues was performed using the avidin-biotin-peroxidase complex method (Ramos-Vara, 2005). Intensities of immunostaining were classified as negative, mild, moderate, and strong, according to Spencer and Bazer (Spencer and Bazer, 1995). Three different slides from each rat were scored per primary antibody and were examined by under 10X, 20X, 40X, and 100X magnifications.

### Proliferating cell nuclear antigen

Identification of mitosis in liver and kidney tissues according to (Hooper, 1961) was performed on paraffin sections mounted on positively charged glass slides. Antigen retrieval was performed in sodium citrate buffer (PH 6.0) (2 cycles of 15 min each). Endogenous peroxidase activity was blocked using 0.05% hydrogen peroxide for 15 min. Non-specific binding was blocked using ultra-violet block. Then slides were incubated overnight at 4°C with primary antibodies against (PCNA) {Catalogue No. MA5-11358 from ThermoFisher Scientific (1:100)}. Biotinylated- secondary antibodies were used, followed by the streptavidin-biotin-peroxidase method. The immunological reaction was visualized using diaminobenzidine. All sections were counter-stained with hematoxylin. The sections were washed with phosphate-buffered saline after each step. Negative controls were included using non-immune serum instead of the primary or secondary antibodies.

### Image analysis for evaluation of immune-histochemical observations

Sections stained with anti-PCNA were analyzed using a digital Leica Quin 500Â image analysis system (Leica Microsystems, Switzerland) housed at the Faculty of Agriculture, Cairo University. The image analyser was calibrated automatically to convert pixels into units of area (µm^2^). PCNA immunostaining was presented as percent of total area in a standard measuring frame over ten independent fields from different slides in each group at 400X magnification. All areas with positive immunohistochemical staining were chosen for evaluation, regardless of the intensity. The mean values and standard deviation obtained for each specimen were analysed statistically.

### Statistical analysis

All quantitative results were analyzed using SPSS version 17.0 for Windows. Data are presented as mean ± SD. Comparisons among multiple group means were performed using a one-way analysis of variance (ANOVA) followed by LSD test. Statistical significance was set at *p* < 0.05.

## Results

### Characterization of NiO-NPs

**FIGURE 2:**
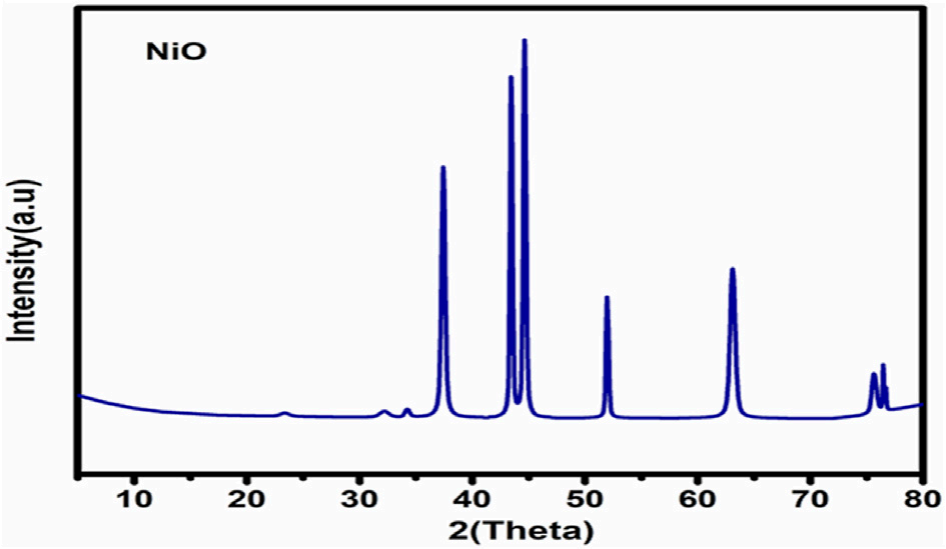
XRD patterns of Nickel oxide nanoparticles.

**FIGURE 3:**
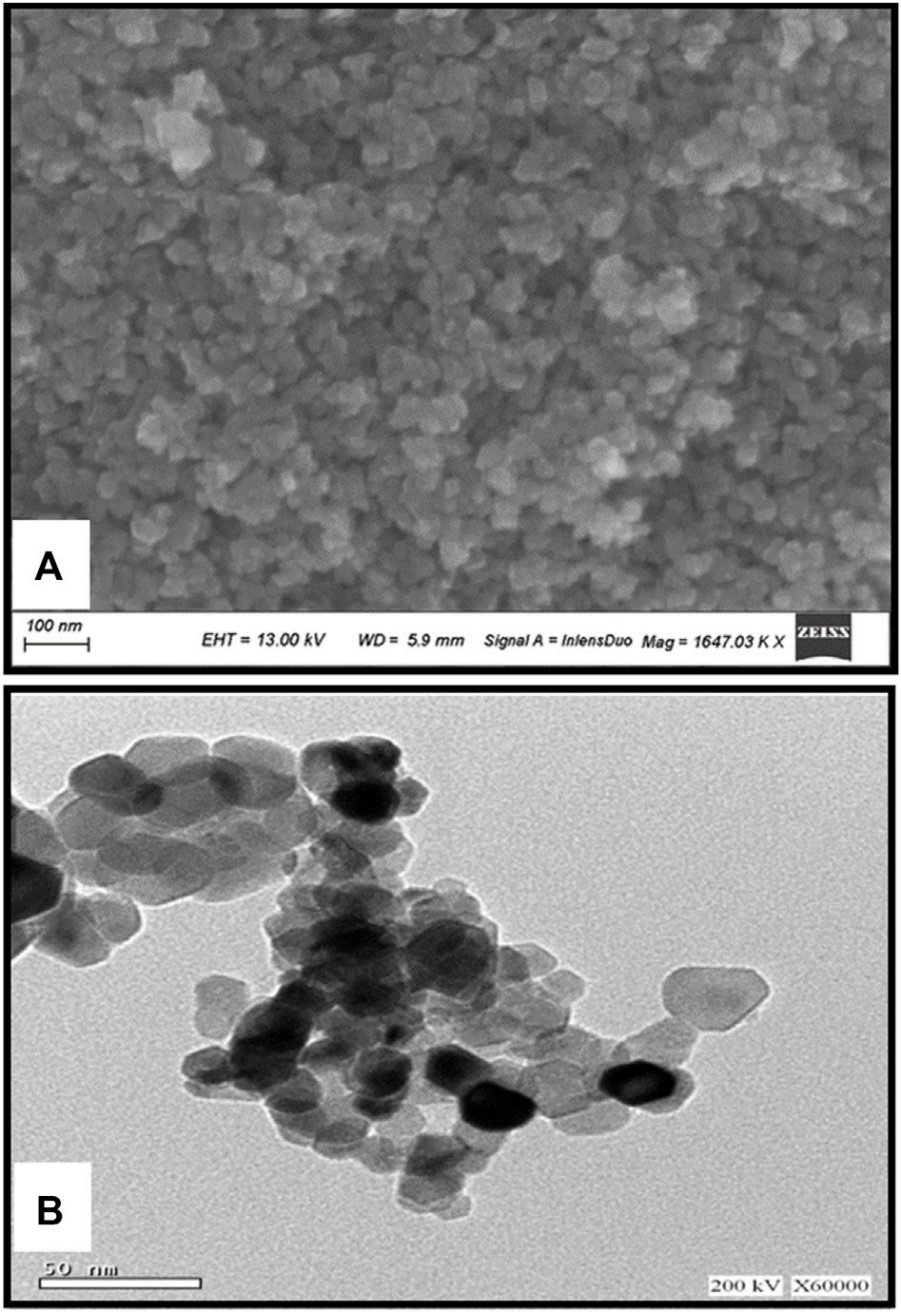
The XRD Difractogram **(A)**; Transmition electron micrograph **(B)**; Scanning electron photomicrograph of Nickel oxide nanoparticles. The XRD reveals the high crystalinity and purity of the prepared powder. The crystallite size is calculated automatically from the XRD softweare to be 42 nm which is confirmed visually from TEM and SEM photomicrographs **(A,B)**.

Figure 2 illustrates the XRD chart patterns of the prepared Nickel Oxide Nanoparticles revealing that NiO-NPs are prepared in pure crystalline phase with an average crystallite size of 42 nm. In good agreement with the XRD Data, Figure 3A shows TEM and Figure 3B shows SEM photomicrographs indicating the homogeneity of size and morphology of the prepared nanoparticles.

### Liver and kidney function parameters

Administration of NiO-NPs resulted in a significant increase in the serum levels of ALT, AST, urea, and creatinine in group II compared to Control group (*p* < 0.05). On the other hand, HSP alone did not cause a significant change in the levels of these biochemical parameters between HSP and Control groups. The coadministration of HSP with NiO-NPs resulted in a significant decrease in the level of ALT in NiO-NPs + HSP group compared to NiO-NPs group (*p* < 0.05), with a non-significant change between the levels of ALT in NiO-NPs + HSP group and Control group. While the levels of AST, urea, and creatinine were significantly decreased in NiO-NPs + HSP group compared to NiO-NPs group (*p* < 0.05), albeit they remained significantly above their levels in Control and HSP groups (*p* < 0.05) (Table 2).

**TABLE 2:** Effects of NiO-NPs and/or HSP exposure on liver and kidney function parameters in the serum of rats.

Group	ALT (U/L)	AST (U/L)	Urea (mg/dl)	Creatinine (mg/dl)
Control	63.53 ± 13.06 ^+^	121.72 ± 16.83 ^+^	19.97 ± 2.52 ^+^	0.49 ± 0.1 ^+^
NiO-NPs	114.91 ± 19.89 *^#^	203.89 ± 21.58 *^#^	37.36 ± 2.93 *^#^	1.03 ± 0.11 *^#^
HSP	59.16 ± 11.93 ^+^	114.77 ± 19.28 ^+^	18.28 ± 2.31 ^+^	0.44 ± 0.07 ^+^
NiO-NPs ^+^ HSP	83.84 ± 16.10 ^+^	154.47 ± 18.76 *^+#^	26.41 ± 2.16 *^+#^	0.69 ± 0.13 *^+#^

Values are presented as mean ± SD (n = 10 rats/group). *: Significantly different from Control group. +:Significantly different from NiO-NPs group. #: Significantly different from HSP group. Statistical significance level at *p < 0.05*.

### Oxidative stress and lipid peroxidation parameters

**FIGURE 4:**
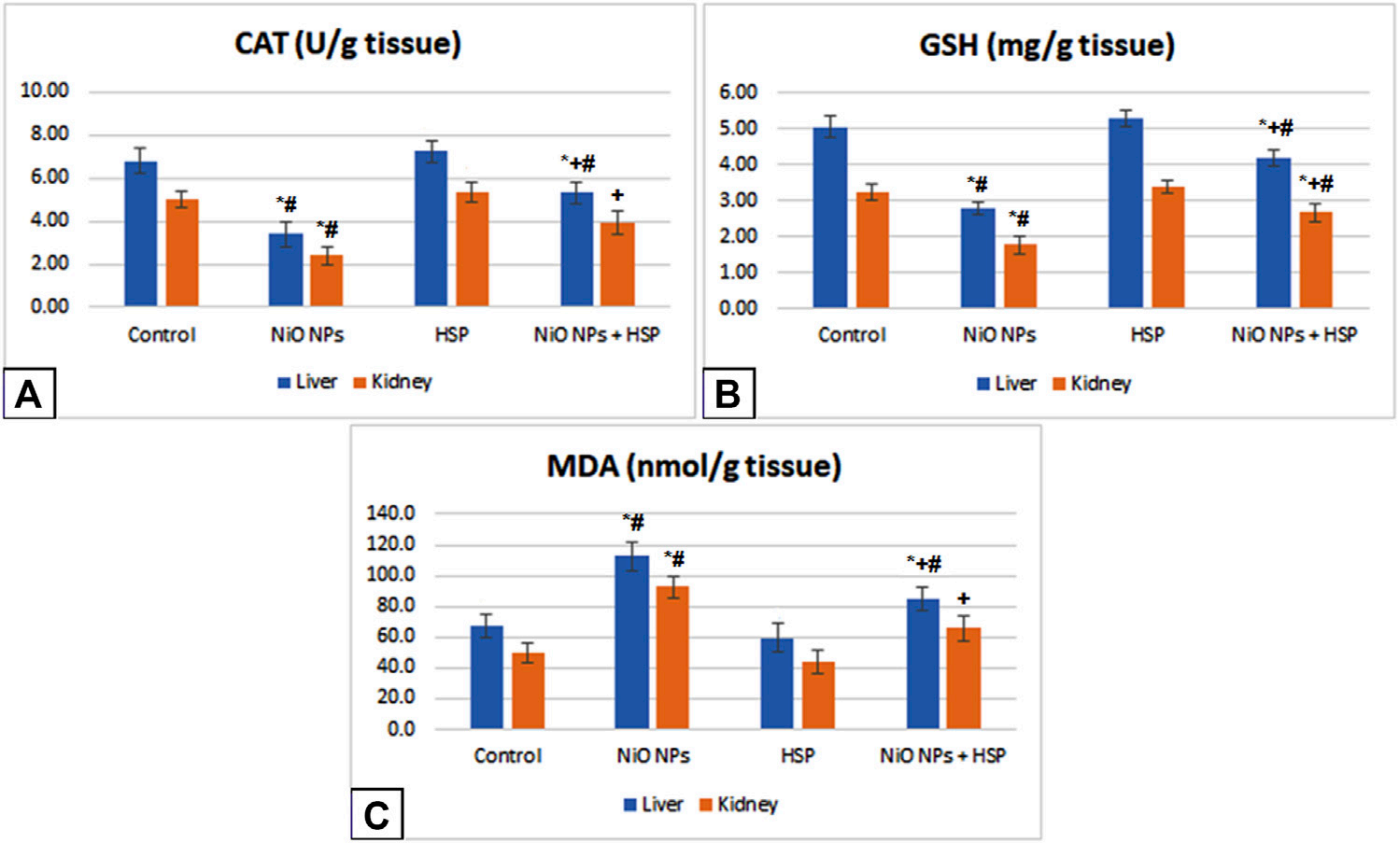
The effects of NiO-NPs and/or HSP exposure on oxidative stress and lipid peroxidation parameters, catalase (CAT, Fig. 4A), glutathione (GSH, Fig. 4B) and malondialdehyde (MDA, Fig. 4C), in the liver and kidney of rats. Values are presented as mean ± SD (n = 10 rats/group). *: Significantly different from Control group. **+**: Significantly different from NiO-NPs group. #: Significantly different from HSP group. Statistical significance level at *p < 0.05*.

Exposure to NiO-NPs induced a significant decrease in CAT enzyme activity and GSH content with a significant increase in MDA content in the liver and renal tissues of NiO-NPs group compared to Control group (*p* < 0.05). Moreover, administration of HSP resulted in a non-significant change in hepatic and renal levels of CAT, GSH, and MDA in the liver and kidney of Group III compared to Control group. However, the concurrent exposure to HSP and NiO-NPs resulted in a significant increase in the hepatic and renal antioxidant levels of CAT and GSH in NiO-NPs + HSP group compared to NiO-NPs group (*p* < 0.05). In addition, MDA content was significantly decreased in the liver and kidneys of the rats exposed to both NiO-NPs and HSP in Group IV compared to NiO-NPs group (*p* < 0.05) **(** Figure 4).

### The transcript levels of Nrf-2, TNF-α, and NF-κB

**FIGURE 5:**
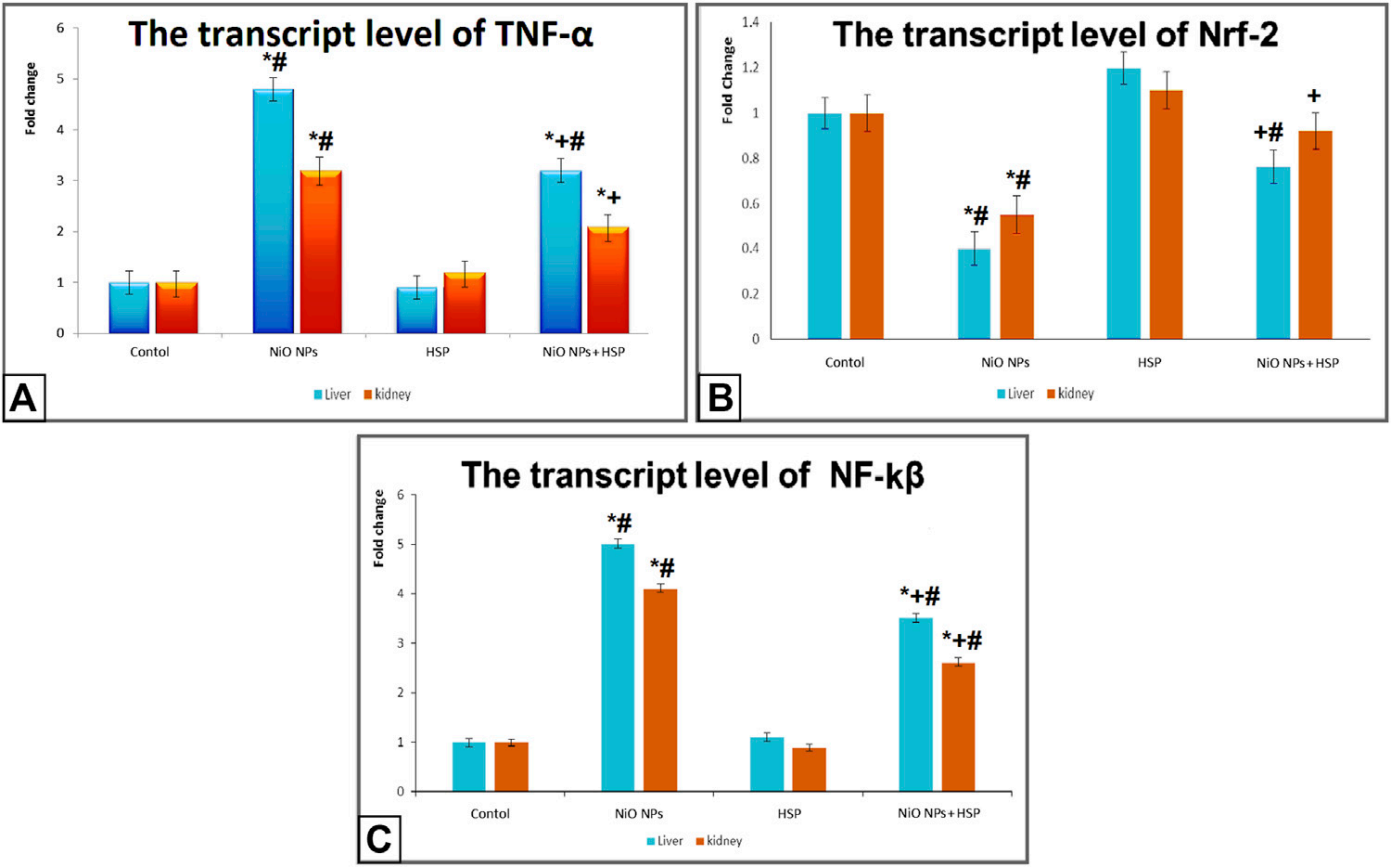
The effects of NiO-NPs and/or HSP exposure on the transcript levels of TNF-α **(Fig. 5A)**, Nrf-2 **(Fig. 5B)**, and NF-*ĸ*β **(Fig. 5C)** in the liver and kidney of rats. Values are presented as mean ± SD (*n* = 10 rats/group). *: Significantly different from Control group. +: Significantly different from NiO-NPs group. #: Significantly different from HSP group. Statistical significance level at *p < 0.05*.

Exposure to NiO-NPs resulted in a significant upregulation of *TNF-α* and *NF-κB* genes in the liver and kidneys of NiO-NPs group compared to Control group (*p* < 0.05). While the concurrent exposure to HSP significantly corrected the genotoxicity induced by the NiO-NPs by decreasing the mRNA levels of both *TNF*-α and *NF*-κB. On the other hand, NiO-NPs caused a significant downregulation of *Nrf*-2 in the liver and kidneys of NiO-NPs group which was modified and increased by the concomitant administration of HSP in group IV (Figure 5).

### The transcript levels of apoptotic markers; *BAX and Bcl-2*

**FIGURE 6:**
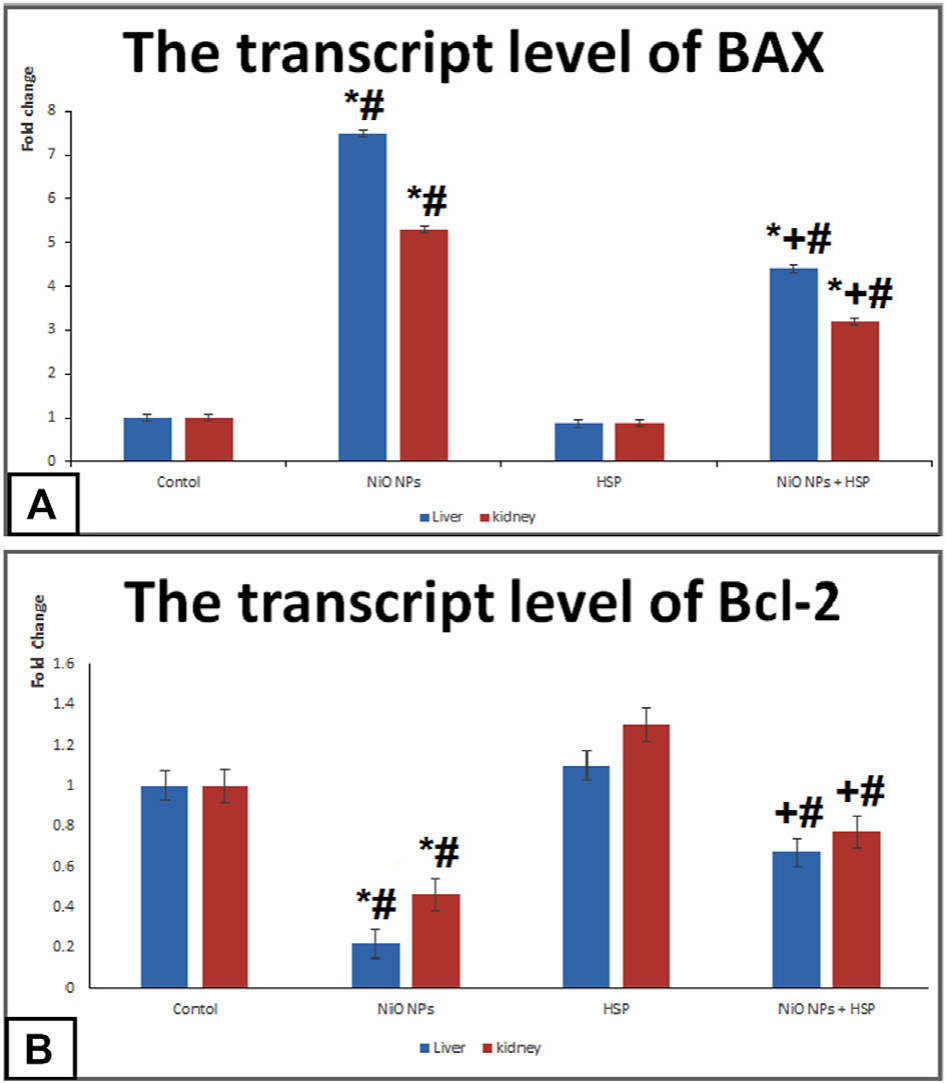
The effects of NiO-NPs and/or HSP exposure on the transcript levels of apoptotic markers; BAX **(Fig. 6A)** and Bcl-2 **(Fig. 6B)** in the liver and kidney of rats. Values are presented as mean ± SD (n = 10 rats/group). *: Significantly different from Control group. +: Significantly different from NiO-NPs group. #: Significantly different from HSP group. Statistical significance level at *p < 0.05*.

Administration of NiO-NPs caused a significant upregulation of BAX apoptotic biomarker in the liver and kidneys of Group II rats compared Control group. On the other hand, NiO-NPs resulted in a significant downregulation of Bcl-2 in the liver and kidneys of NiO-NPs group compared to Control group. Concurrent administration of NiO-NPs with HSP significantly corrected the genotoxicity induced by the NiO-NPs by decreasing the mRNA levels of *BAX* and increasing the levels of *Bcl*-2 in the liver and kidneys of NiO-NPs + HSP group (Figure 6).

### Histopathological examination

**FIGURE 7:**
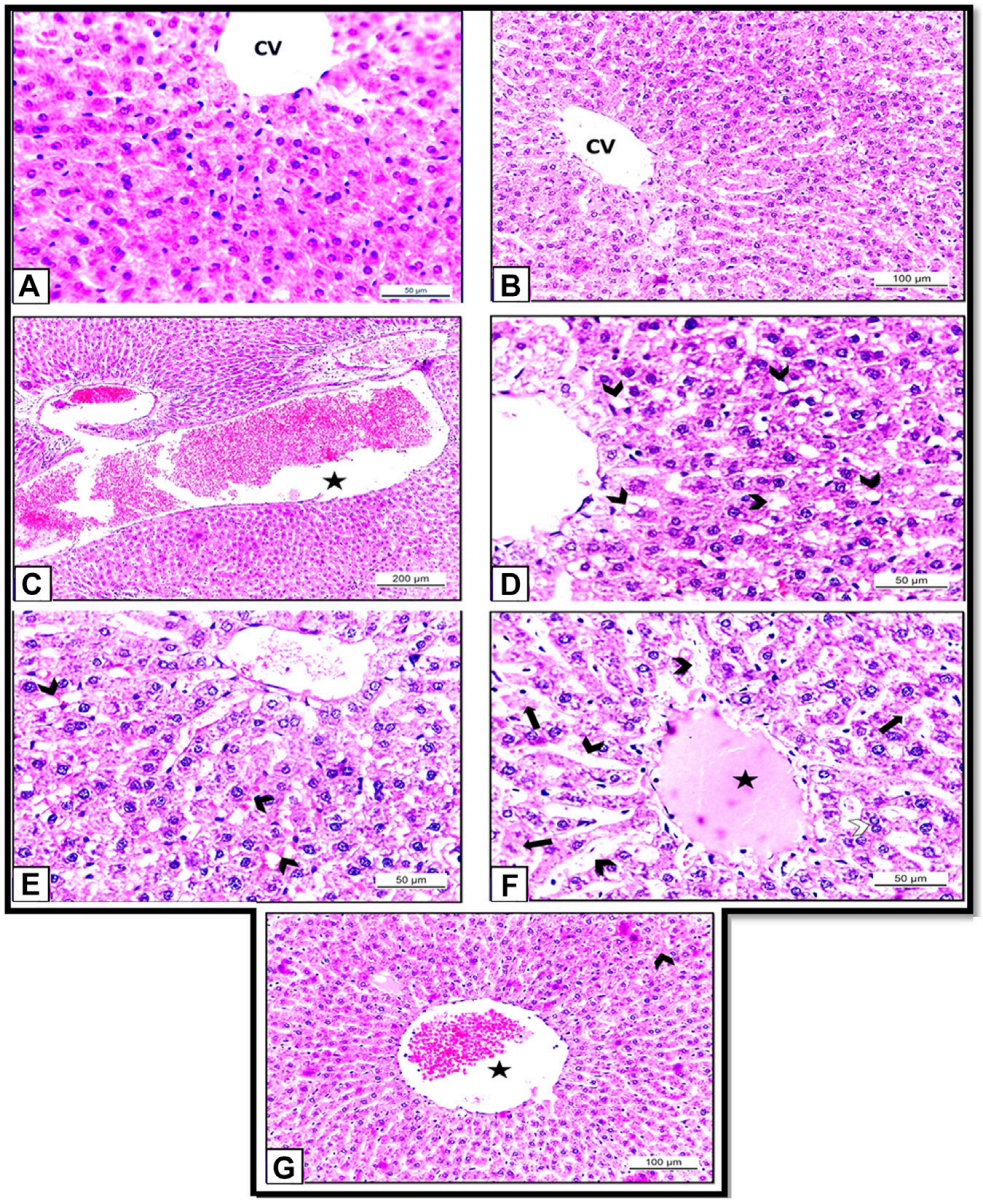
**(A)**: Section of albino rat’s liver of Control group (Group I) showing normal hepatic architecture with central vein and radiating cords of normal hepatocytes with central rounded vesicular nuclei. H&E X400. **(B)**: Section of albino rats’ liver of HSP group (Group III) also showing normal hepatic architecture with hepatic cords separated by blood sinusoids. H&E X 200. **(C–F)**: Sections of albino rats’ livers of NiO-NPs group (Group II). **(C)**: Showing blood vessels are dilated and engorged with blood (star). H&E X100. **(D)**: Showing vacuolar degeneration of the hepatocytes (arrow heads). H&E X400. **(E)**: showing dilated blood sinusoids (arrow heads). H&E X 400. **(F)**: Showing dilated blood sinusoids (arrow heads), hyalinization (star), degenerated hepatocytes (arrows). H&E X 400. **(G)**: Section of albino rat’s liver of NiO-NPs + HSP group (Group IV) showing slightly congested central vein (star) with a decrease in the degenerated hepatocytes (arrow head). H&E X 200.

Histopathological examination of the hepatic tissues obtained from the rats of Control and HSP groups showed normal hepatic architecture with central vein and radiating cords of normal hepatocytes with central rounded vesicular nuclei. Hepatic cords are separated by blood sinusoids (Figures 7A,B). These results supposed that the rats were healthy; and the experiment was conducted under proper conditions.

By contrast, histopathological investigation of the rats that received NiO-NPs (Group II) revealed that the distribution of hepatocytes was scattered and dissociated with loosely arranged hepatocytes. The blood vessels were severely dilated and engorged with blood (Figure 7C). Degenerated hepatocytes with extensive cytoplasmic vacuolation (Figure 7D), dilated blood sinusoids, and hyalinization also could be detected (Figures 7E,F). The degenerated hepatocytes appeared with pyknotic nuclei and vacuolar cytoplasm (Figures 7D,F).

Comparison between Groups II (NiO-NPs) and Group IV (NiO-NPs + HSP) showed that the HSP did not completely revert the damages caused by NiO-NPs, but its administration obviously mitigated the histopathological changes induced by the NiO-NPs. Concomitant administration of HSP in group IV was able to diminish the hepatocyte degeneration and vacuolation and reduce the congestion and hemorrhage compared to the group that were administered NiO-NPs alone (Group II). Also, NiO-NPs + HSP group (Group IV) showed a slightly congested central vein with slightly dilated blood sinusoids. Most hepatocytes appeared normal with acidophilic cytoplasm, and vesicular nuclei (Figure 7G).

**FIGURE 8:**
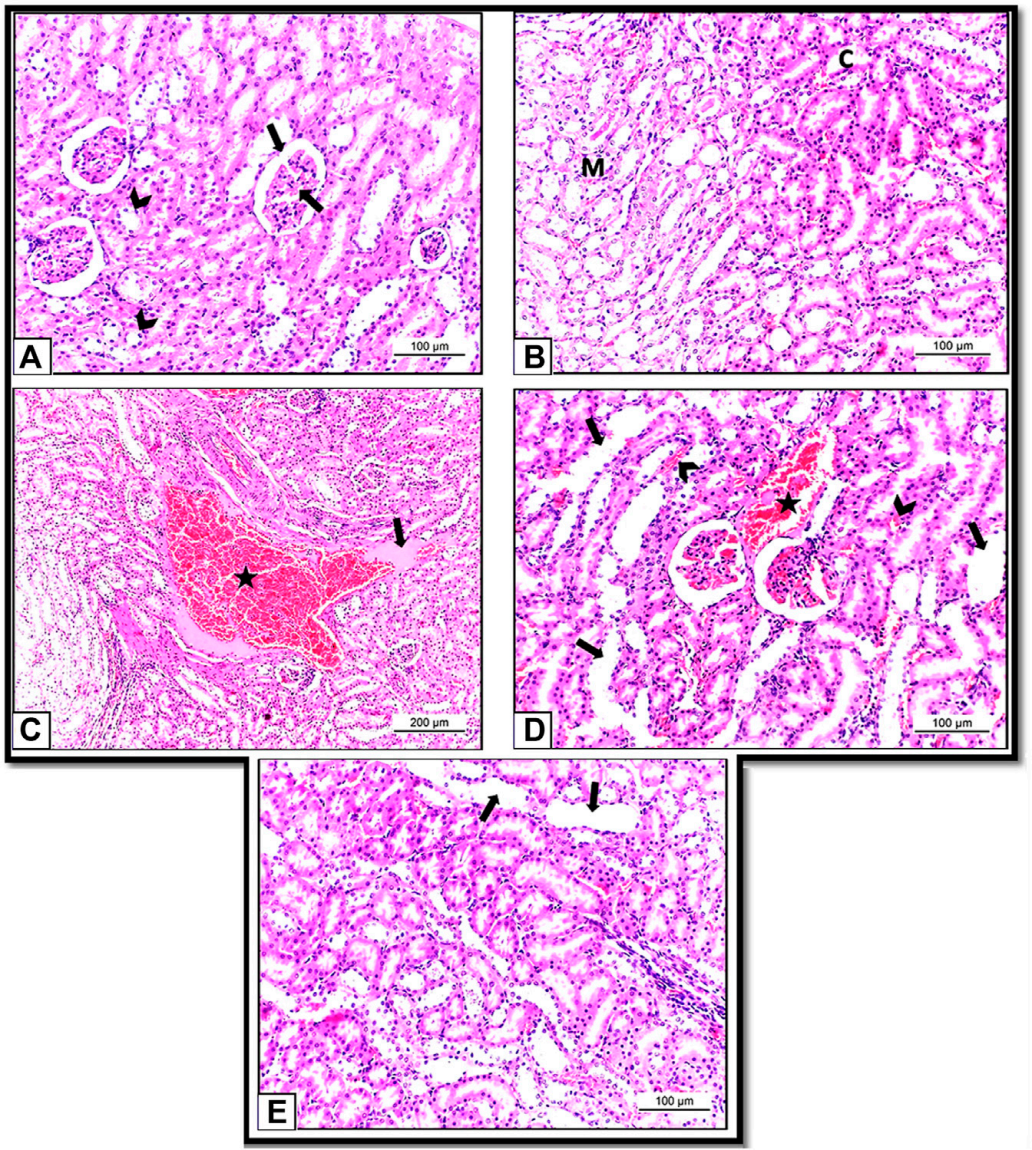
**(A)**: Section of albino rat’s kidney of Control group (Group I) showing normal renal architecture with normal tubules (arrow heads) and glomeruli (arrow). H&E X200. **(B)**: Section of albino rat’s kidney of HSP group (Group III) also showing normal renal architecture of renal tubules in both cortex **(C)** and medulla (M). H&E X 200. **(C,D)**: Sections of albino rats’ kidney of NiO-NPs group (Group II). **(C)**: Showing blood vessels are dilated and engorged with blood (star). H&E X 100. **(D)**: Showing vacuolar degeneration of the hepatocytes (arrow heads). H&E X 200. **(E)**: Section of albino rat’s kidney of NiO-NPs + HSP group (Group IV) showing decreased tubular degeneration and congestion with few degenerated tubules (arrows). H&E X 200.

Histopathological examination of the kidneys of Control and HSP groups showed normal renal architecture with normal tubules and glomeruli (Figures 8A,B). These results supposed that the rats were healthy and the experiment was conducted under proper conditions.

However, kidney sections from NiO-NPs exposed rats (Group II) showed disturbance in the cellular architecture in all renal cells with vast areas of hemorrhage and cast formation (Figure 8C). Tubular epithelium degeneration and desquamation with loss of the brush border and glomerular and tubular congestions were also detected. The degenerated cells appeared with irregular shapes, dark stained cytoplasmic, and condensed nuclei compared to the healthy cells (Figure 8D).

Concomitant administration of HSP with NiO-NPs (Group IV) showed significant restoration of the renal morphology with decreased tubular degeneration and congestion. HSP also showed vascular protection capacity by diminishing the interstitial blood in the kidney (Figure 8E).

### Immuno-histochemical results

**FIGURE 9:**
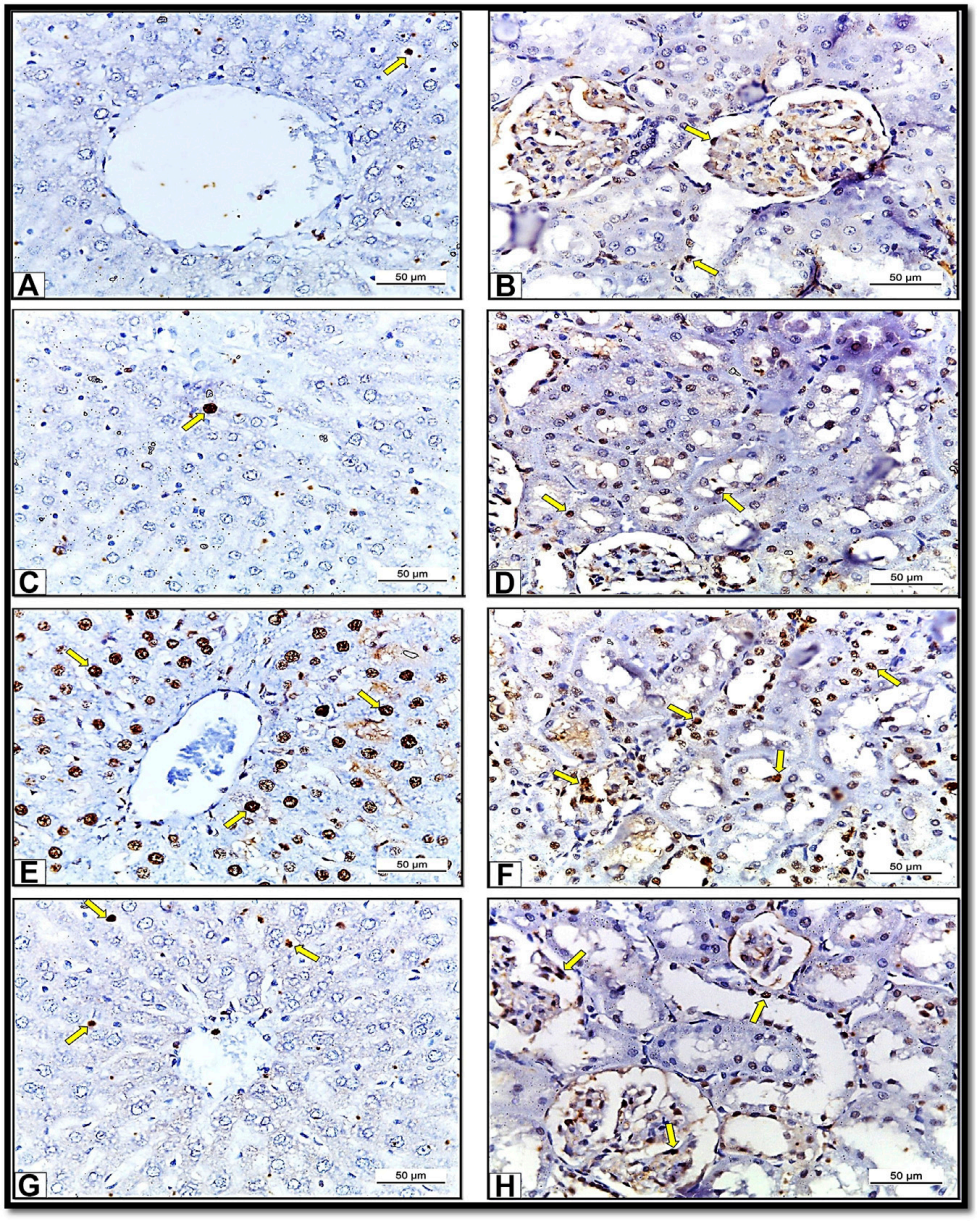
Showing sections of albino rats’ liver and kidney from different groups. Sections of liver **(Fig. 9A)** and Kidney **(Fig. 9B)** of Control group (Group I) showing mild PCNA immunoreactions. X400. Sections of liver **(Fig. 9C)** and Kidney **(Fig. 9D)** of HSP group (Group III) showing mild PCNA immunoreaction. X 400. Sections of liver **(Fig. 9E)** and Kidney **(Fig. 9F)** of NiO-NPs-exposed (Group II) showing strong PCNA immune-reactivity. X400. Sections of liver **(Fig. 9G)** and Kidney **(Fig. 9H)** of HSP+NiO-NPs-exposed (Group IV) showing moderate PCNA immunoreaction. X 400.

The immune-histochemical examination of the sections from the liver (Figure 9A) and kidney tissues (Figure 9B) of Control group (Group I) and liver (Figure 9C) and kidney (Figure 9D) from HSP group (Group III) rats revealed mild PCNA immunereactivity in some areas. In comparison, in the rats exposed to NiO-NPs (Group II), we detected strong PCNA immune-reactivity in all hepatic (Figure 9E) and renal cells (Figure 9F). Finally, the liver (Figure 9G) and kidney (Figure 9H) sections from the rats exposed to NiO-NPs + HSP (Group IV) showed moderate PCNA immune-reactivity.

### Quantitative analysis of the immune-histochemical observations

**FIGURE 10:**
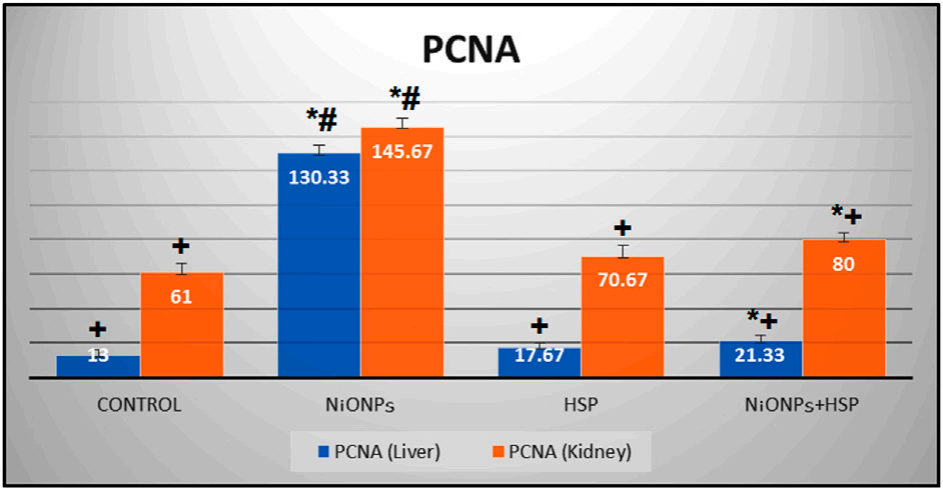
A bar graph showing a significant increase in the number of PCNA-positive immune-reactive cells within the liver and kidneys of NiO-NPs-exposed rats compared to Control and HSP supplemented rats (*p < 0.05*). Coadministration of HSP with NiO-NPs (Group IV) resulted in a significant decrease in the number of PCNA-positive immune-reactive cells compared to NiO-NPs group (Group II) (*p < 0.05*).Data are presented as mean values ±SD. *: Significantly different from Control group. +: Significantly different from NiO-NPs group. #: Significantly different from HSP group. Statistical significance level at *p < 0.05*.

The findings in Table 3 showed a significant increase in the number of PCNA immune-reactive cells in the liver and kidney tissues of the rats exposed to NiO-NPs alone (Group II) compared to the liver and kidney tissues of Control (Group I) and HSP (Group III) rats (*p* < 0.05). Furthermore, co-administration of HSP and NiO-NPs (Group IV) resulted in a significant decrease in the number of PCNA positive immunoreactive cells in the hepatic and renal tissues compared to NiO-NPs group (Group II) (Figure 10).

**TABLE 3:** The effect of NiO-NPs and/or HSP exposure on the number of PCNA-positive immune-reactive cells in the liver and kidneys of rats exposed to NiO-NPs.

Groups	PCNA (liver)	PCNA (kidney)
Group I (Control)	13.00 ± 3.0 ^+^	61.00 ± 5.6 ^+^
Group II (NiO-NPs)	130.33 ± 5.1 *#	145.67 ± 5.1 *^#^
Group III (HSP)	17.67 ± 2.5 ^+^	70.67 ± 6.1 ^+^
Group IV (NiO-NPs + HSP)	21.33 ± 3.5 *^+^	80.00 ± 4.0 *^+^

Values are presented as mean ± SD. *: Significantly different from Control group. +: Significantly different from NiO-NPs group. #: Significantly different from HSP group. Statistical significance level at *p < 0.05*.

## Discussion

Recently, NiO-NPs are used in many industrial and biomedical fields (Mu et al., 2011), (Siddiqui et al., 2012). Occupational exposure to NiO-NPs could cause inflammation and injury to multiple organs and tissues (Phillips et al., 2010). As far as we know, this is one of the pioneer studies in discussing the underlying causes and mechanisms of hepatic and renal toxicities of NiO-NPs in rats as well as testing the protective effects of HSP against NiO-NPs induced liver and kidney toxic effects. In the current study, NiO-NPs were supplied orally since the GIT is the primary portal of entry for NPs into the human and animal bodies, as food and water may contain NPs (Rashidi and Khosravi-Darani, 2011).

The levels of ALT and AST enzymes are crucial indicators for the membrane integrity of hepatocytes. ALT and AST levels in blood were utilized as biomarkers for the direct harmful effect of NiO-NPs on liver tissues. Due to the unique NPs effects, average particle size, crystalline form, specific surface area, aggregation state, and surface charge, the liver is one of the most prominent places for NPs accumulation (Cao et al., 2016)**.**

The current study revealed significant increases in the serum levels of ALT and AST enzymes activities in NiO-NPs group (Group II), indicating the induction of liver injury. The elevated levels of liver enzymes could be attributed to the enzymatic leakage from hepatocyte membranes, as the membranes become more permeable after liver cell injury, and the enzymatic activity rises in blood. Our findings can also be attributable to the opsonization of hepatic tissues, which traps nanoparticles and removes them from the blood (Tavares et al., 2017), and this was consistent with the results of Sidhu et al. (Sidhu et al., 2004)**.**

The efficacy of HSP to dramatically lower the blood levels of liver enzymes (ALT, AST) reflecting its hepatoprotective impact against NiO-NPs hepatotoxicity, and demonstrating its ameliorative properties against liver damage. These findings were harmonious with the results of Elshazly and Mahmoud (Elshazly and Mahmoud, 2014) who found that HSP has a hepatoprotective effect against NiO-NPs toxicity due to its role in the induction of the antioxidant enzymes glutathione peroxidase GSH-Px and glutathione reductase GR, which are two major enzymes involved in the hepatocyte redox balance.

Our results also revealed a significant increase in serum levels of urea and creatinine in NiO-NPs group (Group II), indicating the incidence of renal dysfunction. These findings could be explained by NiO-NPs being biotransformed in the liver, entrapped in the reticular endothelium system, and excreted by the kidneys. The kidney’s glomeruli have 3.5 nm slit holes; as a result, NiO-NPs of this size will concentrate inside the renal tissues, causing renal tubular hyperactivity and alterations in the glomerular filtration permeability throughout the clearance process (Tojo and Kinugasa, 2012). Moreover, due to the increased production of ROS as a result of cytolysis, the produced proteins will be enhanced, increasing creatinine and urea levels. Similar results were published by Dumala et al. (Dumala et al., 2018).

The current study found that HSP provided kidney protection by lowering the serum urea and creatinine levels. Our findings were consistent with those of Des Neves et al. (Pires Das Neves et al., 2004) who found that HSP at dosages of 25 mg/kg reduced the blood urea and creatinine in rats suffering from arsenite-induced nephrotoxicity. HSP can decrease cell damage by stabilizing lysosomes and blocking hydrolytic enzymes (ACP and cathepsin-D) leaking from lysosomes, according to the reversal of hydrolytic enzyme levels during HSP therapy (Khandkar et al., 1996). HSP was also able to repair tissue damage and minimize tubular cell vacuolation and vascular lesions caused by any toxicant due to its antioxidant and anti-inflammatory properties (Balakrishnan and Menon, 2007), (Tirkey et al., 2005)**.**

The current study also showed a significant increase in lipid peroxidation with a significant decrease in GSH and catalase concentrations in the liver and kidney tissues of Group II were detected after exposure to NiO-NPs, demonstrating that NiO-NPs have produced a state of oxidative stress in albino rats. Morsy et al. (Morsy et al., 2016) and Behnam et al. (Behnammorshedi et al., 2015) reported similar results in rats after repeated administration of NiO-NPs.

In the current study, MDA levels were elevated in the liver and kidney tissue homogenates after NiO-NPs exposure, confirming its oxidative stress activity. MDA is a tissue damage and lipid peroxidation (LPO) marker (Aitken and Roman, 2008), and due to the increased membranes’ permeability after cell damage, enzymatic activity was seen in the extracellular fluid and serum; a considerable rise in MDA concentration in the liver and kidney tissues implies an increase in phospholipase activity and excess release of lipid peroxyl radicals (Ali, 2019), (Akhtar et al., 2018). NiO-NPs have previously been proven to produce free radicals and induce oxidative stress (Dumala et al., 2018), (Tsao et al., 2017).

Catalase (CAT) is a crucial antioxidant enzyme protecting the cell from oxidative damage induced by reactive oxygen species (ROS). The reduction in CAT activity in our study suggests that NiO-NPs exposure increased the levels of ROS generation and resulted in tissue damage (Powers and Jackson, 2008). Glutathione (GSH) is the most important antioxidant in the cells, protecting them from oxidative damage caused by reactive oxygen species such as free radicals, lipid peroxides, and heavy metals (Hasan et al., 2016). Exposure to NiO-NPs in the current study, led to depletion in the GSH content in liver and kidney tissue homogenates, suggesting that excessive ROS were created resulting in oxidative stress.

Moreover, GSH, as a non-enzymatic antioxidant, in conjunction with other antioxidant enzymes such as superoxide dismutase (SOD), glutathione peroxidase (GPx), and catalase, are widely known to constitute a significant biological defense system that removes ROS and protects cells and tissues from the harmful effects of oxidative stress (Mohamed et al., 2018). To protect the lipid contents of the cell membrane, GSH combines with the electrophile; 4-Hydroxy-3-nonenal, which develops during lipid peroxidation. GPx and GSH are also involved in the GSH redox cycle, where they convert lipid peroxides into other non-toxic molecules, hence safeguarding the mitochondrial and cell membrane integrity. Furthermore, SOD and CAT can breakdown H2O2 and O2•–, alleviating the oxidative stress, and preserving the cell from damage (Ibrahim et al., 2021).

In terms of oxidative stress indicators, the current study found that HSP restored nearly normal GSH and MDA levels as well as CAT activity in the liver and kidney tissues, demonstrating its antioxidant capacity. These findings were consistent with those of Pari et al. (Pari et al., 2015)**,** who found that HSP may protect against toxic oxidative damage in the liver and kidney tissues, possibly due to its antioxidant characteristics that scavenge free radicals.

NP compounds and NPs provoked both cyto- and genotoxicity induction of oxidative stress, which in turn caused deregulating of apoptosis (Ahmad et al., 2016)**,** inflammation, and DNA damage (Huk et al., 2015)**.** Oxidative stress presents a massive hazard due to mitochondria dysfunction, DNA and protein damage. NiO-NPs induced ROS and upregulated the erythroid-derived 2 related factor-2 (Nrf-2) and Hemeoxygenase-1 (HO-1) genes (Magaye et al., 2014)**.**

Pro-inflammatory cytokines are implicated in hepatorenal toxicities. TNF-α provoked the FasL-induced cell death in the hepatocytes (Lutz et al., 2014)**.** Moreover, TNF-α influentially stimulates the NF-kB, which resolves most anti-apoptotic effects in hepatocytes.

TNF-α generates cytotoxic cascades and apoptotic cell death. Nuclear factor-kappa B (NF-κB) is a protein transcription factor related to immunity, inflammation, and cancer (Baltimore and Discovering, 2009)**.** Activation of NF-kB is done through TNF-α cascade and then activated NF-kB regulated, both negatively and positively, the downstream target gene expression that generates FasL cell death resistance (Luo et al., 2005). HSP may recruit various mechanisms to inhibit the integral NF-κB activation and the HSP-triggered apoptosis (Albensi, 2019).

ROS are involved in the pathogenesis of tissue degeneration. The transcription factor Nrf-2 is the primary cellular mechanism that controls the antioxidant and cytoprotective genes. NiO-NPs produce ROS that impairs the oxidant/antioxidant balance (Muhammad et al., 2019). HSP upregulated the Nrf-2, creating cytoprotective actions against the NiO-NPs. These findings supported the suggestion that HSP has potent antioxidant influences.

Histopathological examination of the liver tissues showed morphological changes in the liver architecture, which revealed that NiO-NPs might have accumulated and induced degeneration. The production of reactive oxygen species has been proposed as an indicator of hepatotoxicity (Shuhendler et al., 2014). It is suggested that oxidative stress induced by nanoparticles may be the principal mechanism of their toxicity (Muñoz and Costa, 2012)**.** Oxidative stress could disturb the antioxidant defense mechanisms of the liver by affecting antioxidant-related enzyme activities (Cao et al., 2016). Free radicals accumulate and exert detrimental effects when the body’s antioxidant capacity can no longer protect the cell from oxidative damage (Zhou et al., 2015). A study by Wang et al. (Wang et al., 2008) on iron and zinc oxide NPs in rats has shown adverse effects on the liver after acute exposure.

Histopathological examination of the kidneys showed that the cellular architecture of the renal tissue was also disturbed because the NiO-NPs might have triggered the intracellular defense mechanism, which may have caused the observed morphological changes. Xu et al. (Xu et al., 2019) reported that the degenerated kidney tubular cells which appeared in the NiO-NPs exposed group have the features of apoptotic cells with irregular shape; darkly stained cytoplasm, and condensed nuclei in comparison to healthy cells, which came in accordance with our results. This indicated that NiO-NPs also have a considerable toxic effect on the kidney tissue.

Our results demonstrated that the significant liver and kidney damage caused by NiO-NPs could be improved after administration of HSP**.** HSP not only protect the hepatic and renal functions but also regress most of the degenerative changes which caused by NiO-NPs exposure. These results suggest that the protective effects of HSP against NiO-NPs induced hepatic and renal toxicities may be through the antioxidant activity of HSP as hydrogen-donors and free-radical scavengers through their potential as chain-breaking antioxidants to inhibit the oxidation of lipoprotein (Cotelle et al., 1996). Furthermore previous studies have indicated that HSP can inhibit the free radical formation and the propagation of free-radical reactions by chelating transition metal ions (Haller and Hizoh, 2004). Antioxidants and drugs of herbal origin proved to be beneficial in reversing hepatic and renal toxicities (Aniya et al., 2005). In addition, HSP administration did not cause any histopathological alterations in liver and kidney tissues when given alone. Several previous studies Pires Das Neves et al. (Pires Das Neves et al., 2004), Arafa et al. (Arafa et al., 2009), Shagirtha and Pari (Shagirtha and Pari, 2011), Trivedi et al. (Trivedi et al., 2011) indicate the protective effect of HSP against other oxidant compounds, including cadmium, doxorubicin, benzo [alfa] pyrene, and sodium arsenite toxicities.

Proliferating cell nuclear antigen (PCNA) is considered an important biomarker of hyper-proliferation (Hall et al., 1990). Detection of PCNA immune-reaction correlated with the changes mentioned above in the liver and kidney tissues induced by NiO-NPs exposure resulted in increased PCNA immune-reactive cells due to increased mitotic activity within the hepatic and renal tubular cells after the NiO-NPs -associated toxic insult. Administration of HSP gradually attenuated the expression of PCNA immune-reactivity in the hepatic and renal tubular cells suggesting its potent anti-hyper-proliferative activity. So, this present study indicates that HSP can limit the number of proliferating cells and shows its protective effect against NiO-NPs induced hepatorenal toxicities. These results were consistent with those previously demonstrated by Siddiqi et al. (Siddiqi et al., 2015).

## Conclusion

The present study has concluded that oral administration of NiO-NPs resulted in obvious damage in rats’ liver and kidney. This damage was evidenced by upregulation of several inflammatory and apoptotic markers such as TNF-α, NF-κB and BAX. Moreover, exposure to NiO-NPs induced oxidative stress and lipid peroxidation and downregulated the expression of different genes like Nrf-2 and Bcl-2 in the rats’ hepatic and renal tissues. Several histopathological alterations and positive expression of PCNA were also noticed in the liver and kidney of NiO-NPs-exposed rats. Most of these harmful effects were reverted or mitigated by the concurrent oral administration of HSP, probably through modulating the enzymatic and non-enzymatic cellular antioxidants, suppressing diverse inflammatory and apoptotic biomarkers, inhibiting the cellular proliferation, and decreasing proliferating cell nuclear antigen (PCNA) which may be the underlying mechanisms of HSP to prevent hepatic and renal toxicities. Therefore, we recommend that dietary supplementation of HSP may be beneficiary in the clinical protection against NiO-NPs exposure, and more research works are required to demonstrate and confirm its efficacy in humans.

## Data Availability

The original contributions presented in the study are included in the article/Supplementary Material, further inquiries can be directed to the corresponding author.
