# Supplementary figures and images for: Hesperidin protects rats’ liver and kidney from oxidative damage and physiological disruption induced by nickel oxide nanoparticles
Source: Front Physiol. 2022 Oct 19;13:912625. doi: 10.3389/fphys.2022.912625 (PMC9626958; doi:10.3389/fphys.2022.912625)

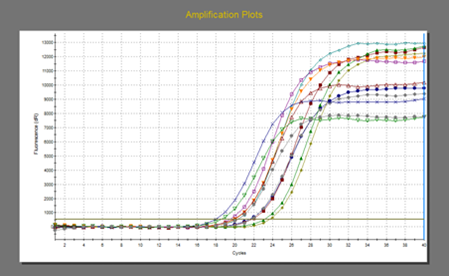

Supplement: Supplementary file 1 [file Image2.PNG]

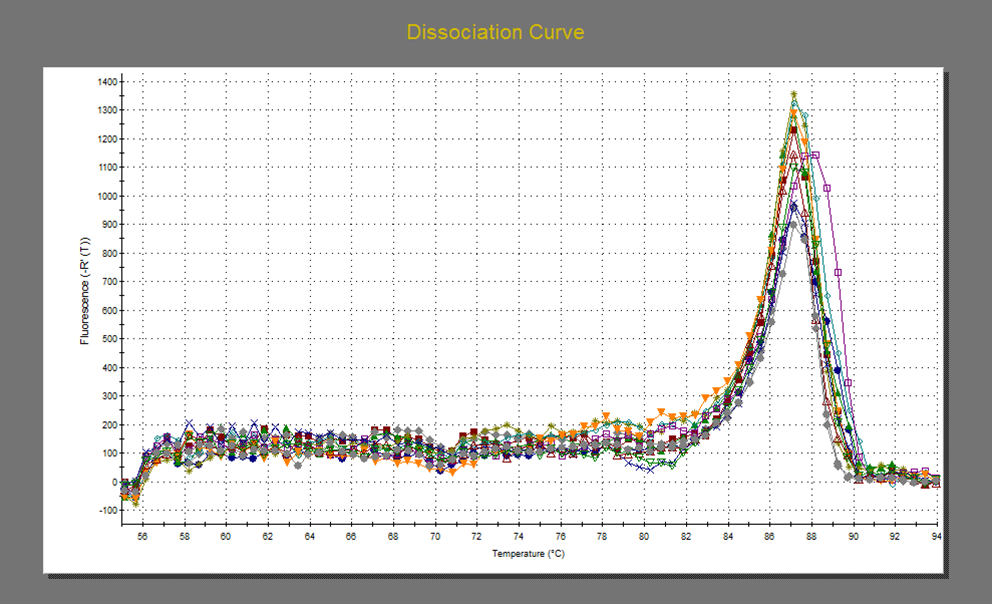

Supplement: Supplementary file 2 [file Image1.PNG]
